# Misdiagnosed for 14 Years: Adenosine Deaminase 2 (ADA2) Deficiency in a Teen Mimicking Polyarteritis Nodosa

**DOI:** 10.1002/ccr3.9641

**Published:** 2024-11-24

**Authors:** Mohammadkian Zarafshani, Masoume Avateffazeli, Seyed Masoud Moeini Taba, Reihaneh Faghihi, Sara Beikmohamadi Hezaveh, Vahid Ziaee, Fatemeh Tahghighi, Maryam Loghman

**Affiliations:** ^1^ Rheumatology Research Center, Shariati Hospital Tehran University of Medical Sciences Tehran Iran; ^2^ Shahid Beheshti University of Medical Sciences Tehran Iran; ^3^ Department of Internal Medicine, School of Medicine, Shahid Beheshti Hospital Kashan University of Medical Sciences Kashan Iran; ^4^ Department of Radiology Kashan University of Medical Sciences Kashan Iran; ^5^ Department of Neurology Arak University of Medical Sciences Arak Iran; ^6^ Pediatric Rheumatology Society of Iran Tehran Iran; ^7^ Children's Medical Center, Pediatrics Center of Excellence Tehran Iran; ^8^ Department of Pediatrics Tehran University of Medical Sciences Tehran Iran; ^9^ Pediatric Rheumatology Research Group, Rheumatology Research Center Tehran University of Medical Sciences Tehran Iran; ^10^ Department of Pediatric Rheumatology, Children's Medical Center Tehran University of Medical Sciences Tehran Iran; ^11^ Department of Rheumatology, Autoimmune Disease Research Center, Beheshti Hospital Kashan University of Medical Sciences Kashan Iran

**Keywords:** autoinflammatory syndrome, autosomal recessive, deficiency of adenosine deaminase type 2

## Abstract

The deficiency of adenosine deaminase 2 (DADA2) is an autosomal recessive disorder caused by loss of function mutations in the ADA2 gene (previously the CECR1 gene) on chromosome 22q11. The clinical spectrum of the disease is remarkably broad, and its presentations mimic features of polyarteritis nodosa, such as livedoid rash, hematological abnormalities (e.g., cytopenia), early‐onset stroke, hypogammaglobulinemia, and systemic inflammation. Early diagnosis and treatment of DADA2 are crucial, as the clinical features could be potentially life‐threatening but treatable. In this study, a 17‐year‐old male patient is reported with DADA2 whose symptoms mimic those of polyarteritis nodosa. A 17‐year‐old male patient presented with a 14‐year history of abdominal pain, hypertension, and cutaneous lesions initially attributed to polyarteritis nodosa (PAN). He was referred to our center due to ongoing abdominal pain. An abdominal and pelvic computed tomography scan with contrast revealed a retroperitoneal hemorrhage compressing the left kidney. Given his history of abdominal pain, hypertension, hemiparesis, transient ischemic attacks (TIA), anemia, cutaneous lesions, and retroperitoneal hemorrhage, DADA2 was suspected, and a genetic test confirmed the diagnosis. Treatment with anti‐TNF (Adalimumab) was initiated, resulting in noticeable improvement. In the follow up, fever, abdominal pain and TIA episodes were subsided and now he has a good clinical condition. Considering DADA2 and conducting a broad screening for other manifestations is recommended for patients presenting with PAN‐like symptoms. These patients may become symptomatic later in life, and early diagnosis allows for the consideration of disease‐specific treatment options.

**Summary**

- Considering DADA2 and conducting a broad screening for patients who present with PAN‐like presentations, especially with ischemic stroke, is crucial.
- In this case, we present a challenging instance of a 17‐year‐old male patient with DADA2 whose symptoms mimic those of polyarteritis nodosa.

## 1 Introduction

Deficiency of adenosine deaminase type 2 (DADA2) is a complex autoinflammatory and autosomal recessive condition characterized by biallelic hypomorphic loss‐of‐function mutations in the adenosine deaminase 2 (ADA2) gene that produces ADA2 [1, 2, 3, 4, 5, 6]. The ADA2 gene, previously identified as the CECR1 (cat eye syndrome chromosome region 1) gene, is located on chromosome 22q11.1 [3, 7]. ADA2 is an enzymatic protein primarily produced by myelocytes, monocytes, and macrophages [3, 4]. It contributes to preserving vascular integrity, regulating neutrophil activity, and exerting anti‐inflammatory effects [3, 7, 8]. DADA2 was initially introduced by Zhou et al. in 2014 as a systemic disorder characterized by vasculitis, neurologic symptoms, hypertension (HTN), skin lesions (purpura, livedo reticularis, and Raynaud's), and hematologic manifestations [2, 3, 9]. Most patients are pediatric, similar to other monogenic autoinflammatory disorders. Since the first case was identified, the number of individuals diagnosed with DADA2 has grown significantly. Some adult patients have not been recognized, as this disorder was identified only a decade ago [10, 11]. The prevalence of DADA2 is estimated to be 1 in 222,164 people worldwide [12]. The main etiology of DADA2 is not yet clear. However, changes in the distribution of M1 and M2 macrophages and disruptions in macrophage differentiation are thought to be implicated. Injury to various tissues results from poor control of neutrophil activity, endothelial cell dysfunction, and increased levels of pro‐inflammatory cytokines [7].

Even among individuals with DADA2, there is a wide range of clinical findings, symptoms, and ages of onset [1, 3]. The majority of the manifestations can be divided into three groups: vasculopathy, hematologic abnormalities, and immunologic abnormalities [3]. The most severe symptoms include cerebral deficits, liver disease, and marrow aplasia, which can be life‐threatening [1].

DADA2 can resemble polyarteritis nodosa (PAN), involving multiple organs and exhibiting characteristics of vasculitis in more than 75% of patients [9, 13]. Several studies on DADA2 have confirmed various phenotypes of this disease, with a considerable number of features overlapping with PAN [9]. Therefore, patients with PAN‐like disease, a family history of PAN, and neurological symptoms accompanied by systemic inflammation, particularly early‐onset strokes, should be evaluated for DADA2. Although the manifestation of DADA2 might be similar to early‐onset PAN, it presents a different phenotype [7].

In this study, we report a 17‐year‐old male patient with a history of PAN‐like disease since childhood, followed by neurologic manifestations and vasculitis.

## 2 Case History/Examination

A 17‐year‐old male patient with a 14‐year history of PAN disease was referred to our center (Children's Medical Center, affiliated with Tehran University of Medical Sciences) in August 2023 with abdominal pain. He complained of colicky, non‐positional pain in the left upper quadrant and left flank. The patient denied any other accompanying symptoms, including fever, urinary frequency, dysuria, nausea, and vomiting.

The patient had a history of abdominal pain, HTN, and cutaneous lesions, including petechiae and Raynaud phenomenon, starting 14 years ago when he was diagnosed with PAN. He had a negative family history of rheumatologic diseases and was treated with prednisolone 2.5 mg/day and azathioprine 50 mg/day. He also experienced a brief hemiparesis on the left side of his body, diagnosed as a transient ischemic attack (TIA), and had an episode of tonic–clonic seizures at the age of 5. Following the seizure, he was treated with carbamazepine 200 mg/day, which was discontinued after a year without any further seizures. The treatment with prednisolone and azathioprine was continued. Additionally, at the age of 15, the patient was admitted with complaints of abdominal pain, anemia, and bloody diarrhea, without any evidence of inflammatory bowel disease.

### 2.1 Differential Diagnosis, Investigations and Treatment

At the age of 17, the patient experienced abdominal pain and was referred for further investigation. During admission, he was alert and oriented. His vital signs were as follows: blood pressure of 160/100 mmHg, heart rate of 83 beats per minute, respiratory rate of 15 breaths per minute, oral temperature of 36.5°C (97.7°F), and oxygen saturation of 96% in ambient air. Clinical evaluation revealed skin color alterations and posterior nasal discharge; other examinations were normal. The laboratory test results are shown in Table 1.

**TABLE 1:** Patient's laboratory findings at the admission.

Test	Value	Reference range	Test	Value	Reference range
White blood cell (/μL)	11,600	4500–11,000	Urine analysis	Normal	Negative
Neutrophil (%)	67	45–75	AST(U/L)	16	10–40
Lymph (%)	22	16–46	ALT (U/L)	6	10–55
RBC (/μL)	3800	4.4–5.8	ALK‐P (U/L)	312	45–115
HB (g/dL)	9.2	13–18	CPK (μg/L)	92	10–120
HCT (%)	29.9	37–49	Angiotensin converting enzyme (nmol/mL/min)	17.9	< 40
MCV (μm^3^)	78	78–100	ESR (mm)	93	< 20
Platelets (× 10^3^/μL)	590	130–400	CRP (mg/dL)	96	< 5
Creatinine (mg/dL)	0.7	0.8–1.3	ANA	0.22	
BUN (mg/dL)	16	8–25	P‐ANCA (EU)	0.5	< 1.4
Uric acid (mg/dL)	4.3	3.5–7.2	C‐ANCA (EU)	1.1	0.00–0.22
LDH (U/L)	651	50–150	HLA B5	Positive	Negative
Creatinine (24 h urine) (mg/day)	689	500–2000	HLA B51	Positive	Negative
Protein (24 h urine) (mg/day)	117	< 150	HBS Ag (mIU/mL)	0.17	< 5

**FIGURE 1:**
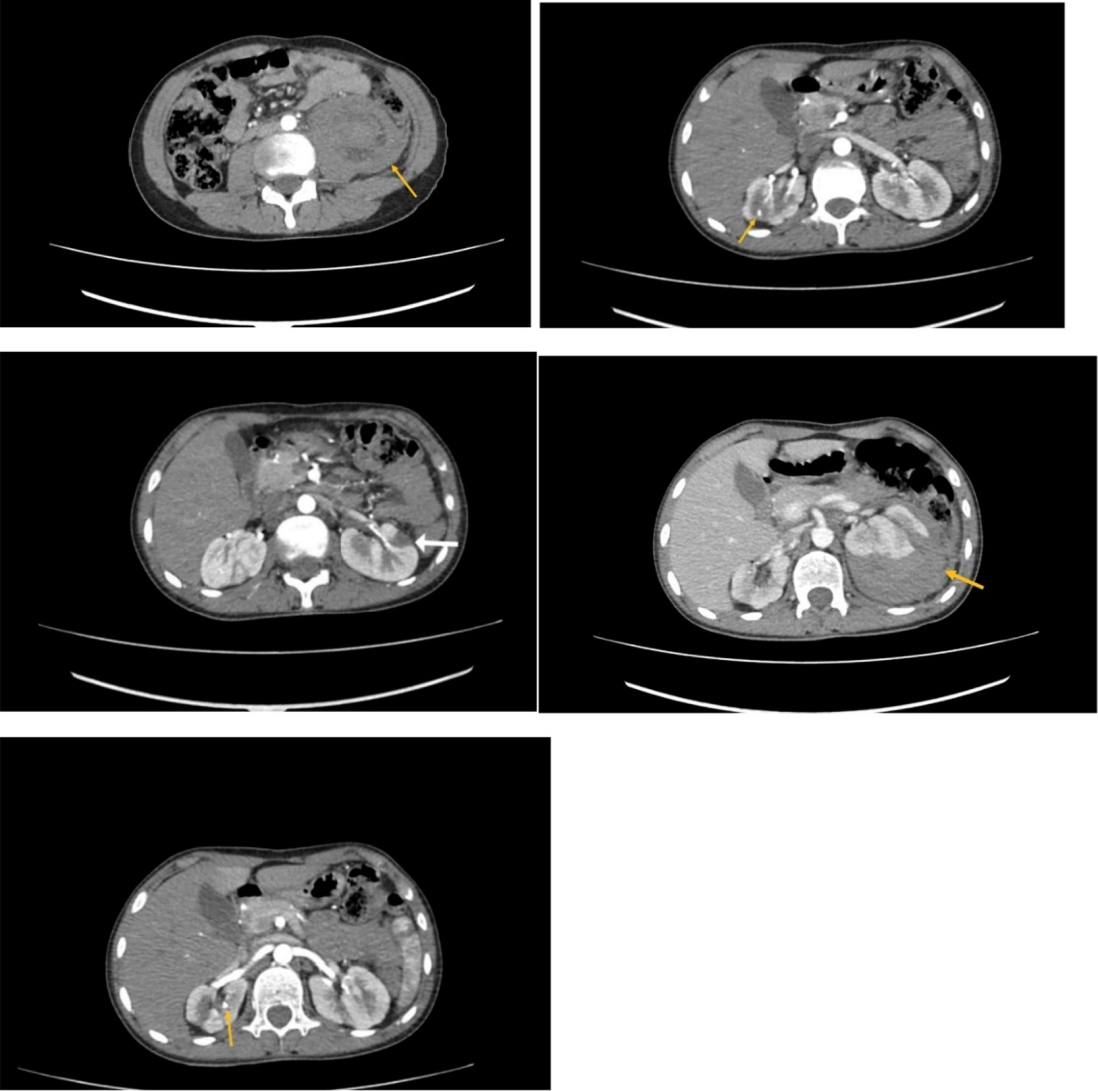
Axial CT scan of abdomen with retroperitoneal hemorrhage with compression of left kidney previous infarct and no evidence of aneurysms.

**FIGURE 2:**
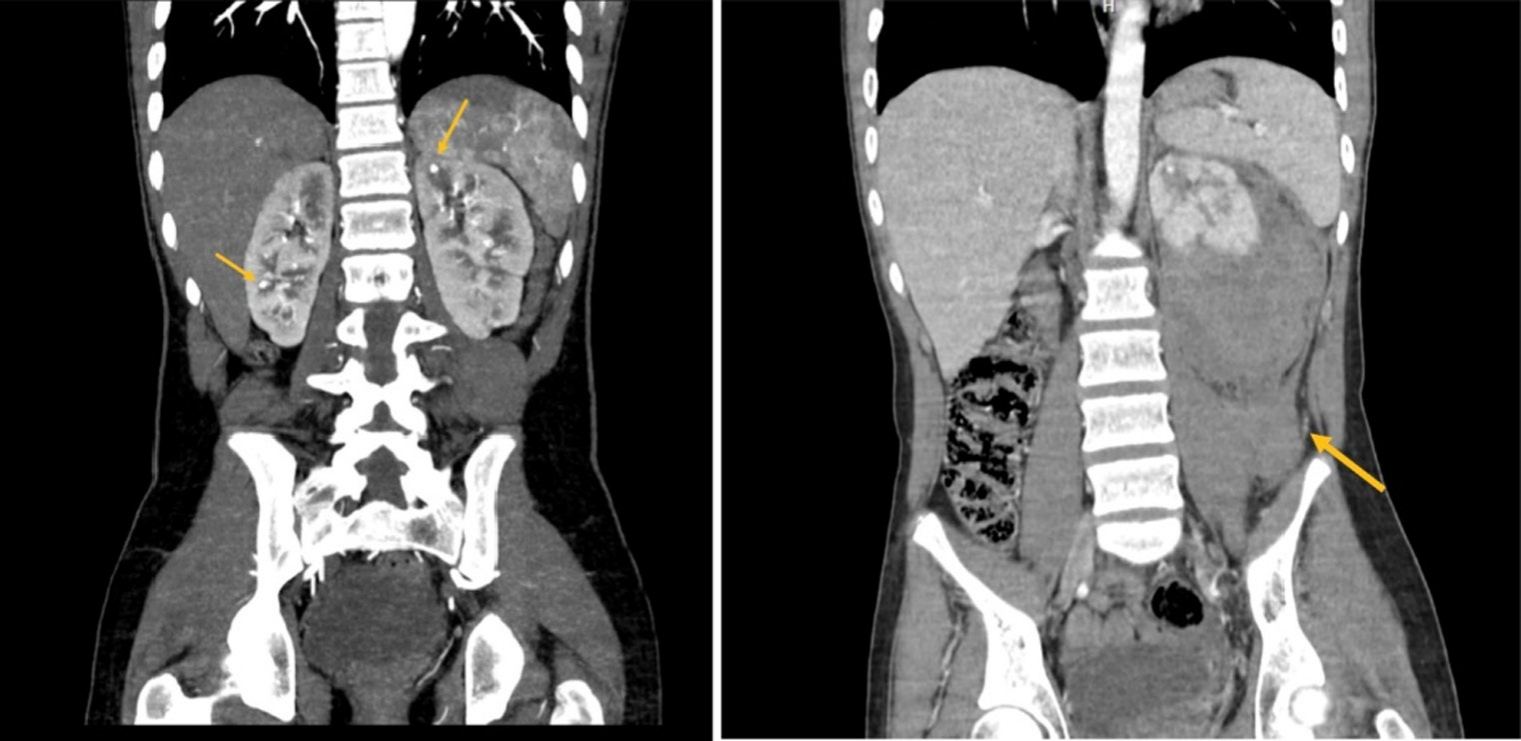
Coronal CT scan of abdomen with previous infarct.

An abdominal and pelvic computed tomography (CT) scan with contrast during his admission revealed a retroperitoneal hemorrhage with compression of the left kidney and a hetero‐dense, non‐enhancing area in the left kidney, compatible with previous infarcts and hematomas. Notably, this CT scan, like the brain CT scan from his childhood, showed no evidence of aneurysms (Figures 1 and 2). The patient's symptoms significantly improved after receiving one pulse of cyclophosphamide 750 mg and 3 days of methylprednisolone 500 mg/day. Additionally, maintenance therapy with azathioprine 75 mg/day and prednisolone 50 mg/day for 1 month was continued, with the prednisolone dosage reduced to 25 mg/day as his overall condition improved.

Given the combination of symptoms and findings, including abdominal pain, retroperitoneal hemorrhage without aneurysms, HTN, cutaneous lesions, hemiparesis, TIA, and anemia, the diagnosis of DADA2 was suspected. Therefore, a genetic test was performed to confirm the diagnosis. Next Generation Sequencing results showed an autosomal recessive (AR) homozygous mutation ADA2: p.T360A [rs775440641], while his parents were heterozygous. The results for tuberculosis (TB), interferon‐gamma (IFN‐Gamma), hepatitis C (HCV), hepatitis B (HBV), and human immunodeficiency virus (HIV) were negative. Furthermore, immunoglobulin M (IgM), immunoglobulin E (IgE), and immunoglobulin A (IgA) levels were below the normal range. As a result, prednisolone and azathioprine were tapered, and treatment with anti‐TNF (adalimumab) was initiated.

### 2.2 Outcome and Follow‐Up

During the follow‐up, fever, abdominal pain, and TIA episodes were broken down, leading to a significant improvement in his condition.

## 3 Discussion

In this case report, we describe a 17‐year‐old male with DADA2 who had been misdiagnosed with PAN for nearly 14 years. The patient received prednisolone and azathioprine, with increased dosages for each flare‐up, before he was definitively diagnosed with DADA2. Initially, he presented with HTN, cutaneous lesions (petechiae and Raynaud phenomenon), hemiparesis, TIA, retroperitoneal hemorrhage, abdominal pain, anemia, and elevated liver function tests (elevated alkaline phosphatase). These findings, particularly the lack of aneurysm evidence in the last admission, suggested reconsidering the diagnosis.

DADA2 is a monogenic and autosomal recessive disease. The mutation can result in ADA2 protein loss of function and decreased ADA2 plasma activity [14]. The disease's characteristics are diverse, with a wide range of features identified, including vasculopathy, skin lesions, CNS involvement such as early‐onset stroke, ischemic stroke, hemorrhagic stroke, and TIA, mild hypogammaglobulinemia, and hematologic manifestations like anemia since childhood [3, 4, 5, 13, 15]. Besides these symptoms, HTN and gastrointestinal involvement, including abdominal pain, have been reported in several patients [1, 9, 16, 17]. Vasculitis affecting small and medium‐sized arteries is the cause of the clinical spectrum of DADA2 syndrome [5]. According to Lee et al., DADA2 patients can be divided into three main groups: pure red cell aplasia (PRCA), bone marrow failure (BMF), and vasculitis. Vasculitis is the most common category of DADA2 [18]. Furthermore, it can mimic other diseases, including PAN, as in this case. Genetic analysis is strongly recommended in individuals with early‐onset PAN diagnosis, vasculopathy, and end‐organ damage, particularly stroke [4, 13]. Vasculitis can present as HTN and GI manifestations [16]. Cutaneous involvement can present as livedo reticularis/racemosa, nodules, purpura, Raynaud's, and erythema nodosum [9]. The CNS and cutaneous involvement are the most frequently affected tissues. Although CNS manifestation is infrequent in PAN (5% of cases), it is common in DADA2 (50% of cases) [13]. In more than half of the patients, central nervous system symptoms were reported. This patient experienced TIA and one seizure episode, along with hemiparesis, which was notable at the time of his last admission. Ischemic strokes or TIA can occur in infants as young as 5 months of age [1]. Therefore, various neurologic manifestations, including intracranial hemorrhage, neuropathies, cranial neuropathy, stroke, and even seizures, can be observed [7].

Intestinal stenosis and/or aneurysms of the mesenteric, celiac, and renal arteries have been observed on angiography in certain individuals, with necrotizing vasculitis found on intestinal histology [19]. However, the CT‐angiography examination in the present case did not show any signs of aneurysm or artery stenosis, similar to the findings of Yin et al. This might occur because the small arteries implicated in DADA2 are not often sensitive enough for CT and MRA to detect [16].

Laboratory evaluations can show anemia, elevated liver function tests (LFTs), inflammatory markers (ESR and CRP), and negative levels of ANA, P‐ANCA, and C‐ANCA, as seen in our patient [3, 13, 15].

In this study, we depicted the features of our patient compatible with vasculitis and the PRCA phenotype. DADA2 syndrome is thought to be a rare condition. However, it is crucial to assess patients with neurologic and systemic symptoms as soon as possible to prevent subsequent complications [8]. Based on previous studies, DADA2 is more than just a rheumatologic condition, and variability in the manifestations can delay the diagnosis of DADA2, as occurred in our case. Therefore, it must be discussed with hematologists, neurologists, and immunologists to start appropriate and timely therapy [3]. Early recognition and proper treatment are necessary to reduce the high rate of mortality and morbidities in DADA2, as the mortality rate is higher in DADA2 (18.7% in DADA2 vs. 2.9% in PAN of pediatric patients) [1, 10].

The optimal treatment for DADA2 patients is still controversial. Prescribing anti‐tumor necrosis factor (TNF) agents (etanercept, infliximab, and adalimumab) in patients with vasculitis phenomena, including strokes, seems to be the cornerstone of DADA2 treatment and reduces levels of inflammatory markers [1, 3, 9, 15]. Anti‐TNF‐alpha therapy has been shown to effectively manage flare‐ups, and prompt treatment with these medications can improve inflammation and control the progression of the disorder. However, the length and frequency of the therapy are not yet clear [5].

## 4 Conclusion

Considering DADA2 in patients with ischemic stroke and conducting a broad screening of other manifestations is recommended for patients who present with PAN‐like presentations. Because of its variable phenotype, patients with DADA2 may present to a hematologist, immunologist, neurologist, pediatrician, or gastroenterologist rather than to a clinical rheumatologist. Therefore, a multidisciplinary approach to DADA2 patients is necessary.

Anti‐TNF agents are considered the mainstay of treatment for DADA2 patients, whereas bone marrow transplants are emerging as the definitive treatment for the hematological phenotype and may also be effective for immunological manifestations and anti‐TNF refractory cases. Gene therapy may be a promising future treatment and pave the way for better outcomes in this devastating disease.

## Author Contributions

**Mohammadkian Zarafshani:** writing – original draft, writing – review and editing. **Masoume Avateffazeli:** data curation, writing – review and editing. **Seyed Masoud Moeini Taba:** formal analysis, writing – review and editing. **Reihaneh Faghihi:** data curation, writing – review and editing. **Sara Beikmohamadi Hezaveh:** formal analysis, writing – review and editing. **Vahid Ziaee:** formal analysis, writing – review and editing. **Fatemeh Tahghighi:** formal analysis, writing – review and editing. **Maryam Loghman:** supervision, writing – review and editing.

## Ethics Statement

Written informed consent was obtained from the patients in our study. The purpose of this research was completely explained to the patient and they were assured that their information will be kept confidential by the researcher. The present study was approved by the Medical Ethics Committee of the academy.

## Consent

Written informed consent was obtained from the patient for publication of this case report and any accompanying images. A copy of the written consent is available for review by the Editor‐in‐Chief of this journal.

## Conflicts of Interest

The authors declare no conflicts of interest.

## Data Availability

All data regarding this study has been reported in the manuscript. Please contact the corresponding author if you are interested in any further information.
